# The Impact of Two MMPI-2-Based Models of Personality in Predicting Driving Behavior. Can Demographic Variables Be Disregarded?

**DOI:** 10.3390/brainsci11030313

**Published:** 2021-03-02

**Authors:** Luigi Tinella, Alessandro Oronzo Caffò, Antonella Lopez, Ignazio Grattagliano, Andrea Bosco

**Affiliations:** Department of Educational Sciences, Psychology, Communication, University of Bari, 70121 Bari, Italy; alessandro.caffo@uniba.it (A.O.C.); antonella.lopez@uniba.it (A.L.); ignazio.grattagliano@uniba.it (I.G.); andrea.bosco@uniba.it (A.B.)

**Keywords:** fitness-to-drive, personality, cognition, regression models, assessment, MMPI-2

## Abstract

The driver’s personality is a key human factor for the assessment of the fitness to drive (FTD), affecting driving decisions and behavior, with consequences on driving safety. No previous study has investigated the effectiveness of Minnesota Multiphasic Personality Inventory (MMPI)-2 scales for predicting the FTD. The present study aimed to compare two MMPI-2-based models of normal and pathological personality traits (i.e., Inventory of Driving-related Personality Traits (IVPE)-MMPI vs. Personality Psychopathology Five (PSY-5) scale) in predicting the cognitive FTD. One hundred young and eighty-seven adult active drivers completed the MMPI-2 questionnaire as a measure of personality and a computerized driving task measuring for resilience of attention (Determination Test (DT)), reaction speed (Reaction Test (RS)), motor speed (MS), and perceptual speed (Adaptive Tachistoscopic Traffic Perception Test (ATAVT)). The effects of age, gender, and education were also controlled. Results showed that the models controlled for demographics overperformed those neglecting them for each driving outcome. A negative effect of age was found on each driving task; the effect of gender, favoring males, was found in both the RS and the MS, and the effect of education was found on the DT and the ATAVT. Concerning personality traits, significant effects were found of sensation seeking (IVPE-MMPI) on each outcome; of anxiety (as a measure of emotional instability; IVPE-MMPI) and introversion (PSY-5) on the measures of MS; and of psychopathic deviation (as a measure of self-control; IVPEMMPI) on the DT. The study confirmed the key role of demographic factors in influencing the FTD, further suggesting the usefulness of some MMPI2-based personality scales in the assessment of driving-related personality determinants.

## 1. Introduction

Research on traffic accidents shows that the human factor is considered the main factor underlying motor vehicle crashes [1,2,3,4,5]. Driving a motor vehicle is a complex activity that relies on visual, motor, and cognitive skills [6,7,8,9]. The other psychological factor that plays a key role in driving performance is personality, as it affects both driving behaviors and the likelihood of being involved in car accidents [10,11]. Personality can be defined as the individual difference in the tendency to show consistent patterns of thoughts, feeling, and actions [11,12]. According to Nichols et al. (2012), “Personality significantly affects much of what we do as humans” [13] (p. 134). The way in which the individual personality has an impact on driving behavior could be considered a relevant issue to road safety [14,15]. In fact, several scholars agree on recognizing the driver’s personality as a more general or distal factor that interacts with more proximal factors (e.g., driving skills and driving behaviors), thus determining the likelihood of being involved in motor vehicle accidents [10,16,17,18].

Despite the wealth of literature in the field, according to Sommer et al. [5], it seems to be extremely difficult to determine a clear relationship between personality, driving behaviors, and accidents. According to Poó and Ledesma [15], such complexity seems to be attributable to the heterogeneity of variables that affect driving behaviors, such as drivers’ characteristics, behavioral patterns, and the environmental context [3], which have been studied separately rather than in a multidimensional approach [19]. 

Moreover, personality traits have been assessed using different theoretical models (e.g., the five-factor model or the sixteen personality factors), which makes it challenging to integrate and compare results [15]. Thus, the research on driving-related personality traits is rather fragmented [20]. 

Personality traits seem to influence driving goals and driving performance, which are two components of the tactical level of the hierarchical model of driving behavior proposed by Michon [6] as being composed by three levels: strategic, tactical, and operational. 

The strategic level includes decisions and choices that precede the driving activity (e.g., travel destination, the vehicle, and the road to go through). At the tactical level, the driving goals and driving performance are adjusted according to real traffic demands by cognitive processes and decisions. The operational level describes the implementation of driving maneuvers and reactions to the driving environment, supported by cognitive and motor resources. 

Thus, driving-related personality traits differentially affect the three levels of the driving behavior model. In this context, the present study addresses the relationship of both driving-related and pathological personality traits with cognitive prerequisites for the fitness to drive. The following sections highlight the main findings of the different frameworks of personality traits investigated in driving research. Moreover, the links between normal and pathological personalities, the driver’s demographic characteristics, and cognitive prerequisites for driving are also introduced. 

### 1.1. Models of Personality Traits in Driving

The relationship between personality and the fitness to drive has been usually examined following different theoretical frameworks such as the five-factor model [21], the Myers–Briggs Type Indicator [22], Eysenck’s personality model [23], the alternative five-factor model [24], and Cattell’s sixteen personality factors [25]. All these personality perspectives are suitable for studying driving-related personality traits, and they are associated with certain driving measures. 

The five-factor model [21,26,27] is the best-accepted personality theory, and it is composed of neuroticism, extraversion, openness, agreeableness, and conscientiousness. Extraversion has been shown to be positively associated with risky driving [28,29,30]. Neuroticism seemed to be related to vehicular accidents and fatalities [30,31,32]. Conscientiousness was found to be negatively correlated with risky driving [33] and motor vehicle crashes [34]. Concerning agreeableness and openness, the former was shown inversely associated with risky driving [33], whereas the latter was positively associated with traffic accidents [35].

Regarding Eysenck’s theory of personality [36], which classifies personality into extraversion, neuroticism, and psychoticism, high scores on extraversion have been found to be positively correlated with motor vehicle crashes and aberrant driving behaviors [37,38]. Neuroticism was associated with aggressive driving and fatigue [39,40]. Finally, psychoticism showed a positive correlation with self-reported driving behaviors [41] and driving skills [42]. 

By using Cattell’s sixteen personality factors (16PF) [43], previous studies have demonstrated that [44] measures of emotional stability, liveliness, warmth, social boldness, abstractedness, openness, and dominance are combined with risky driving and a higher motor vehicle crash incidence [44,45,46]; on the other hand, measures of vigilance, apprehension, self-reliance, perfectionism, sensitivity, and tension have shown associations with safe driving [44,45,46]. Moreover, Yan et al. [47] clustered the 16PF scores obtaining four personality types, namely inapprehension, insensitivity, apprehension, and unreasoning. The participants labeled within the unreasoning type had the worst simulated driving performance, with more collisions than the participants belonging to the other three types. 

Since personality may change during a lifetime [48], influencing in a different way the driving behavior of male and female drivers [49,50], some demographic aspects (i.e., age and gender) need to be considered in the study of driving-related human factors [51]. 

### 1.2. Age and Gender Differences in Driving-Related Personality Traits

The background research approaching driving-related personality traits suggests significant age and gender differences among drivers [51]. The age group most frequently involved in road accidents is represented by young, novice drivers. The young have the tendency to underestimate traffic risks, to overestimate their driving skills, and, finally, to use unsafe strategies to manage hazardous driving events [52,53,54]. According to Zicat et al. [55] sensation seeking, trait anxiety, and anger are considered the personality factors most involved in driving actions among young adults. Sensation seeking has been defined as “the seeking of varied, novel, complex and intense sensations and experiences, and the willingness to take physical, social, legal, and financial risks for the sake of such experience” [56] (p. 27). 

Sensation seeking is strictly linked to impulsivity, but they are not the same construct [57]. While the former refers to the search for stimulating experiences, impulsivity refers to the lack of self-control in response inhibition [57,58]. Young drivers with high sensation seeking were more likely to go fast, to not wear safety belts, to drive after drinking alcohol, to have a low risk aversion, to drive recklessly, to state high self-reported risky driving behaviors, and, thus, to report a high accident risk [59,60,61,62,63,64]. Trait anxiety seemed to have a non-linear relationship with risky driving in young drivers [55]. The high level of trait anxiety was positively associated with risky driving behaviors, while a low anxiety score was more likely associated with a decreased self-perceived crash risk and negative driving attitudes [14,65]. Finally, driving anger seemed to predict aggressive as well as risky driving, and it has been associated with reckless driving behaviors in younger drivers [19,59].

On the contrary, the literature regarding older drivers has devoted less attention to driving-related personality traits focusing mainly on perceptual and cognitive risk factors [9,11,66,67]. According to a recent review [67], studies focusing on standard tools for assessing personality traits (non-driving-specific) generally found no significant associations between them and driving measures. Conversely, studies that employed specific tools for assessing driving-related personality traits (e.g., sensation seeking, anxiety, impulsivity, etc.) have shown strong relationships between them and both rule violations and driving errors [66,68]. Moreover, in the study of Lucidi et al. [9], sensation seeking positively affected self-reported driving violations, whereas hostility showed associations with driving lapses and errors in a sample of active older drivers. Furthermore, anxiety, normlessness, and hostility predicted attitudes toward driving safety, which, in turn, predicted self-reported aberrant driving behaviors [9]. Personality is a key aspect of the fitness to drive in younger drivers, and since personality traits may change during the lifetime (including aging) [48], the relatively few studies on older drivers have argued that it is surprising that the previous literature paid so little attention to the study of personality [11,66]. 

According to Norris et al. [69] and Constantinou et al. [70], the significant effect of gender on car crash proneness might interact with personality among young drivers. Males seemed to show a higher level of sensation seeking, with more self-reported accidents and driving violations than females, who, in turn, presented higher scores on sensitivity to punishment, which may then be considered a protective factor against risky driving behavior [70]. Scott-Parker et al. [71] showed that a high sensation-seeking propensity and owning a car significantly predicted self-reported speeding in young female drivers. Moreover, females were showed to be more likely engaged in self-regulatory behaviors than males [50]. Otedal and Rundmo [49] showed that male drivers, instead, are found to be more likely associated with both normlessness personality and risky driving. Eduardo and Ildefonso [72] found a marked significant effect of personality on traffic accidents in males, while they found no evidence for females. According to the authors, such difference is attributable to gender-related differences in positive affect, risk perception, driver violence, and impulsivity [73,74,75]. 

### 1.3. Normal and Pathological Personality Traits Related to the Cognitive Fitness to Drive

Relatively few studies have investigated the relationship between normal or pathological personality traits and cognitive functions related to driving. By using a driving-specific computerized tool (e.g., the Vienna Test System) [76], Nechtelberger et al. [77] recently investigated the relationship between personality traits, cognitive abilities, and fluid intelligence in drivers with a revoked driving license due to intoxicated driving (alcohol/drug consumption). Authors found that participants with higher emotional instability showed lower hand–eye coordination, poorer selective attention, and poorer resilience of attention, as well as lower perceptual and motor speed, compared with those lower in emotional instability. Moreover, a high level of sense of responsibility was linked to a high fitness to drive, hand–eye coordination, and perceptual speed. Finally, a high level of self-control was found to be a significant predictor of higher motor speed. Vetter et al. [78] found a similar effect of social responsibility on cognitive prerequisites of the fitness to drive in a sample of professional drivers.

Sommer et al. [5] investigated cognitive and personality predictors of driving fitness. The authors measured driving-related abilities and personality traits of participants (e.g., sensation seeking, social responsibility, accepted level of risk, self-control, and emotional stability) and observed their on-road driving performance. First, results showed that the relationship between personality and the fitness to drive may not be linear; indeed, the artificial neural network provided superior results when compared with classical multivariate methods. Second, measures of emotional stability, social responsibility, and the subjectively accepted level of risk added incremental validity to driving-related cognitive abilities in predicting the on-road driving performance. 

Both studies [5,77] used the Inventory of Driving-related Personality Traits (IVPE) [79], which is a standardized questionnaire consisting of 39 items measuring four driving-related personality traits, namely sensation seeking, social responsibility, self-control, and emotional stability. Results from both studies pointed out the predictive role of social responsibility, emotional stability, and self-control on both cognitive prerequisites for the fitness to drive and the on-road driving performance. 

Finally, recent studies have reported age and gender differences in cognitive functions implied in the fitness to drive, such as memory, perceptual speed, motor reaction times, resilience of attention, executive functions, and visuospatial skills [77,80,81].

Regarding pathological personality, according to Alavi et al. [42], mental illness might adversely affect cognitive functions essential for driving, particularly in the case of a comorbidity with alcohol and/or drug abuse [82,83]. The authors stated that the consequences of mental illness could jeopardize the driving behaviors of the normal population. Drivers who reported depression and anxiety and those with psychiatric disorders (e.g., bipolar disorder, schizophrenia spectrum, major depressive disorder) showed an impaired fitness to drive, and they reported significantly higher driving violations and motor vehicle accidents when compared with healthy participants [42,84,85,86]. 

According to Waller [87], the psychopathological condition and its drug therapy leads, at all ages, to impairments in cognitive functioning (e.g., attention and concentration) and psychomotor skills that are determinants to the fitness to drive [84,87]. Mood disorders have been previously associated with fatal car accidents [88] and with decreased reaction times in a simulated driving task [89]. During an on-road driving test, untreated depressed patients showed a higher standard deviation of the lateral position (i.e., a measure of vehicle control tracking) than healthy controls [90]. Furthermore, both young and adult drivers with ADHD (characterized by inattention, hyperactivity, and impulsivity) [91] showed lower simulated driving performance than controls [92], and ADHD was significantly associated with traffic accidents [93]. Anxiety disorders were associated with driving violations and intoxicated driving [94,95,96]. Obsessive-compulsive symptoms increased the odds of accidents by more than twofold in a sample of Persian bus and truck drivers [42]. Moreover, other research [97,98,99] demonstrated that people with personality disorders (e.g., schizoid, paranoid, compulsive, avoidant, borderline, etc.) showed higher accident rates, drunken driving, reckless driving, failure to stop, and improper turns when compared with their controls.

Since psychopathological personality traits may adversely affect cognitive and motor skills involved in driving fitness [84], the assessment and detection of pathological traits in the non-clinical driving population might be essential from a preventative perspective [42]. It is likely that a diagnostic interview employed in the evaluation of a pathological personality and mental illness might help to better estimate the individual fitness to drive, especially for professional drivers [42]. To this purpose, the Minnesota Multiphasic Personality Inventory (MMPI) [100] is one of the most used personality questionnaires for a comprehensive assessment of both healthy and pathological personalities [101]. Despite this, it has been rarely used in driving research. The second version of the tool, MMPI-2 [102], is a 567-item questionnaire that provide scores on 77 personality scales. Among these scales, the Personality Psychopathology Five (PSY-5) scale can also be scored [103]. According to the Personality Psychopathology Five model [104], five broad personality traits (e.g., aggressiveness, negative emotionality, psychoticism, disconstraint, and introversion) are relevant in everyone’s daily living and describe individual differences in personality traits that could impact clinical problems. This model of personality addresses personal differences in adaptive functioning in both clinical and non-clinical samples [105]. 

To the best of our knowledge no previous study has investigated the usefulness of the PSY-5 scale in discriminating the drivers’ fitness-to-drive. 

### 1.4. The Present Study

Psychopathological personality traits might have consequences in daily activities, leading to clinical problems. From a preventative point of view, the screening of these traits in the evaluation of the fitness to drive could be a promising way to discriminate potential risky drivers. Moreover, the focus on driving-related personality traits may be helpful in designing training interventions improving safety in driving practices.

The main aim of the present study was to compare two MMPI-2-based models of personality traits (driving related and pathological) in predicting the measures of cognitive and motor prerequisites of the fitness to drive in a sample of non-clinical young (18–34 years) and adult (37–64 years) drivers. A secondary aim of the study was to investigate the potential effects of the two personality models on driving prerequisites controlling for demographic characteristics (e.g., age, gender, and level of education). To this purpose, the model of traits from the Inventory of Driving-related Personality Traits (IVPE; e.g., sensation seeking, social responsibility, self-control, and emotional stability) [79] was subsumed by selecting associated personality traits provided by the MMPI-2. This model was compared with the Personality Psychopathology Five model (PSY-5; e.g., aggressiveness, negative emotionality, psychoticism, disconstraint, and introversion) [105] in the prediction of resilience of attention, motor speed, reaction speed, and visual perceptual speed, separately.

First, as observed in recent studies [77], a significant and negative effect of age was expected on measures of the fitness to drive in both models. Moreover, a gender effect was expected on measures of resilience of attention in favor of females and on motor speed in favor of males [81]. No effects of education were expected on driving measures. To the best of our knowledge, no previous studies have investigated the employability of PSY-5 personality traits in predicting driving measures. Significant results are expected for an inverse relationship of aggressiveness, psychoticism, and neuroticism on measures of the fitness to drive [30,31,32,84]. In light of previous studies that employed the IVPE [5,77,78], it was hypothesized that (a) sensation seeking would negatively affect all prerequisites of driving fitness due to a decreased ability to adapt responses to the task’s demands [106] and (b) social responsibility, emotional stability, and self-control would positively affect resilience of attention, motor and reaction speed, and perceptual speed independently of age and gender [5,77].

The two investigated MMPI-based models were tested twofold for each outcome: in one instance considering only personality traits and secondly by adding to those demographic variables (i.e., age, gender, and level of education).

## 2. Materials and Methods

### 2.1. Participants

A power analysis to estimate the sample size was carried out using G*Power 3.1 [107] with the following parameters: test family (*F*-tests); linear multiple regression (fixed model, R^2^ increase as a statistical test); a *p*-value of 0.022 (according to a Bonferroni correction/which assumes a mean correlation coefficient of 0.30); a cautious low effect size (0.10); a power of 0.80; and eight tested predictors. Results indicated that a sample size of 162 participants was adequate to warrant an 80% chance of correctly rejecting the null hypothesis. 

This study enrolled 187 healthy participants, including 64 females, between 18 and 64 years of age, of which 100 were younger adults between 18 and 34 years of age (33 females, age mean ± SD = 23 ± 3.53 years, level of education mean ± SD = 13.2 ± 1.03 years) and 87 were mature adults aged between 37 and 64 years (31 females, age mean ± SD = 54.3 ± 7.32 years, level of education mean ± SD = 11.2 ± 2.82 years). Descriptive statistics for the two groups are reported in Table S1. All participants were required to (i) speak Italian as their mother tongue; (ii) hold a valid current driver’s license, provisional or above; (iii) have normal cognitive functioning (above the standardized cutoffs); (iv) have normal or corrected-to-normal vision; (v) have driven more than one time within the last month; and (vii) not be and (viii) not have been a professional driver (e.g., of a taxi, truck, or bus). The participants were volunteers recruited with the support of a proxy informant, generally undergraduate and graduate students, and trainees. All participants were blind to the hypothesis of the study and signed their informed consent prior to enrollment. 

The Ethical Committee of the Department of Education, Psychology and Communication of the University of Bari approved the study protocol, and the whole study was performed following the Declaration of Helsinki and its later amendments.

### 2.2. Materials and Procedures

All participants were from the metropolitan area of Bari, Italy. Participants had to be in a good general state of physical and psychological health. All participants were enrolled between April and July 2019. A brief interview was administered by supervised trainees in clinical psychology and neuropsychology to collect demographic information, to exclude neurodegenerative and vision/acoustic disorders, and to gather information about each participant’s driving rates. After completing the interview, all participants completed the below-described tests.

#### 2.2.1. Overall Cognitive Functioning

The Montreal Cognitive Assessment (MoCA) [108] was used to evaluate the overall cognitive functioning. MoCA assesses several cognitive domains, namely visuospatial/executive, naming, attention, language, abstraction, memory, and orientation, on a 30-point scale. The best cutoff used in an Italian sample was a MoCA score of <=17 [109,110] for discriminating participants with probable cognitive impairment. No one was excluded from the sample.

#### 2.2.2. Fitness-to-Drive Screening

The Fitness to Drive Screening (DRIVESC-Version 03) [77] is a test set of Schuhfried’s Vienna Test System. This tool allows one to validly assess an individual’s fitness to drive [111,112,113,114,115]. The apparatus includes an ergonomic response panel, two foot pedals, a standard audio output device (headset), and a video screen. The experimental screening took approximately 25 min, and it included three subtests: Reaction Test (RT), Determination Test (DT), and Adaptive Tachistoscopic Traffic Perception Test (ATAVT). 

The RT measures the participant’s motor time and reaction time. Participants had to react to a specific combination of auditory and visual stimuli as quickly and as accurately as possible. Each participant was instructed to keep one finger on a rest button while stimuli were presented. When the relevant combination occurred, the participant had to release the finger from the rest button, quickly press the response button, and then immediately replace the finger on the rest button. The RT provides two distinct measures: (i) reaction speed (RS), that is, the time taken to initiate the movement, and (ii) motor speed (MS), that is, the time taken to press the response button after the finger has been lifted from the rest button. The measures are taped in milliseconds: a short time of reaction (i.e., high visual and motor reaction speed) corresponded to a higher ability to quickly respond.

The DT provides a measure of resilience of attention, that is, reactive stress tolerance. Participants had to react as quickly and as accurately as possible to rapidly changing acoustic and visual stimuli of different frequencies and colors. The software varied the speed of stimuli presentation to adapt to each participant’s ability in terms of accuracy (i.e., hits, omissions, and false alarms) and response delay (i.e., milliseconds), providing a unique score. 

The ATAVT measures the ability to gain an overview of a traffic scenario that includes the quick detection of objects and visual patterns [116]. The ATAVT was administered in the right-hand traffic form according to the Italian traffic laws. In this subtest, pictures of everyday traffic scenarios were presented very briefly after an acoustic cue. Participants had to state which of the five groups of objects (i.e., motorcycles/bicycles, automobiles, traffic signs, traffic lights, and pedestrians) they perceived. The participants’ performance was the number of correct responses (omissions and false alarms were also recorded). 

The scores of the three subtests were provided as a raw score (called parameters) and percentile ranks. For statistical analysis, only percentile ranks were considered.

The entire procedure was made clear to the participants beforehand. Participants were assessed individually in a silent and well-lit room, without disturbances, in the Department of Educational Sciences, Psychology, Communication of the University of Bari. Each assessment session was accomplished by two instructed research assistants and lasted 60–90 min, with breaks provided, as requested.

#### 2.2.3. Personality Assessment

The Minnesota Multiphasic Personality Inventory 2 [102] is a personality questionnaire used for the evaluation of the personality profile [117,118]; this tool is one of the most used in the assessment of normal and pathological personalities. It includes 567 true-or-false items. The assessed personality traits are divided into different groups of scales, namely validity, content, clinical, and supplementary indicators. The scores are provided as raw scores and T-scores. According to the Italian validation [118], a T-score of >65 represents attributable symptoms due to the presence of psychopathology. For the purposes of the present study, the personality traits of interest were extracted from different scales and were considered for statistical analysis.

The Personality Psychopathology Five traits were scored from the entire pool of items of the MMPI-2, following the study of Harkness et al. [103]. The PSY-5 scale includes traits of aggressiveness (AGGR), psychoticism (PSY), constraint (DISC), negative emotionality/neuroticism (NEGE), and introversion (INTR).

The IVPE-MMPI-based personality traits were selected according to their shared characteristics with the constructs included in the IVPE (e.g., sensation seeking, emotional stability, self-control, and social responsibility). First, the MMPI-based sensation-seeking scale (MSS) was developed from the item pool of the MMPI-2, inspired by the study of Viken et al. [119], who previously developed and validated an 18-item MMPI-derived sensation-seeking scale. A preliminary factorial analysis was performed on the 16 items available in the MMPI-2. The anxiety (ANX) scale was selected from the group of supplementary traditional scales of the MMPI-2 as a measure of emotional stability. The scale of psychopathic deviation (PD) extracted from the group of clinical scales was selected as a measure of self-control, according to the definition provided in the Manual of the MMPI-2 [120]. Finally, the scale of social responsibility (SR) was selected from the supplementary additional scales from the MMPI-2, mirroring the IVPE’s social responsibility factor. 

### 2.3. Statistical Analysis

A chi-square test between gender (females and males) and age (young and adult) was performed to test for their independence. Independent samples *t*-tests were performed to test differences in performance measures, level of education, and personality measures between both age and gender groups. Factor analysis was performed on the items of the developed MMPI-based sensation-seeking scale (MSS) using Jamovi software [121]. Internal consistency was determined using Cronbach’s alpha. Explorative factor analysis (EFA) was carried out to achieve the best factorial solution for the MMPI-based sensation-seeking scale (MSS), namely a single-factor solution found by Viken et al. [119]. Correlation coefficients and related *p*-values were calculated between all the employed variables. Finally, to investigate the influence of demographic and personality variables on driving performance, a series of multiple regression analyses was performed using the lavaan package in R software [122]. The value of *p* was set to 0.05 for calculation of statistical significance. Two groups of regression models for each driving prerequisite were tested for both PSY-5 vs. IVPE-MMPI-based predictors: (1) one ignoring demographic variables and (2) the other including demographic variables as predictors. The effect size Cohen’s f2 was estimated for each model [123]. For each of the two comparisons (PSY-5 vs. IVPE-MMPI), the Akaike Information Criterion (AIC) was considered. The lower the model’s AIC, the better the model’s fit [124].

## 3. Results

### 3.1. Descriptive Statistics and Preliminary Analyses

Means, standard deviations, and correlation coefficients for the variables employed in the study are showed in Table S1. Cronbach’s alpha is reported for psychometric standardized tools. The chi-square test on contingency tables showed that in the composition of the sample, genders were equally represented in each age group *(χ*^2^ = 0.143, *p* = 0.70). The results of Welch’s *t*-test for independent samples revealed a significant difference in the level of education between young and adult drivers (education: Welch’s *t*(106) = 6.34, *p* < 0.001) with a large effect size (Cohen’s *d* = 0.98), in favor of young drivers. Moreover, the t-test for independent samples revealed significant differences between the age groups in cognitive and driving tests, in favor of young drivers (MoCA: *t*(185) = 3.44, *p* < 0.001; DT: Welch’s *t*(150) = 10.50, *p* < 0.001; MS: *t*(185) = 5.71, *p* < 0.001; RS: *t*(185) = 4.47, *p* < 0.001; ATAVT: t(185) = 6.18, *p* < 0.001). Regarding the measures of personality, the young drivers showed significantly higher scores on sensation seeking and psychopathic deviation (MSS: *t*(185) = 5.85, *p* < 0.001; PD: Welch’s *t*(184) = 3.68, *p* < 0.001) and lower scores on social responsibility (SR: *t*(185) = −2.09, *p* < 0.038) than adult drivers. Considering gender differences, males showed significantly lower motor and reaction times (MS: *t*(185) = −6.87, *p* < 0.001; RS: *t*(185) = −3.05, *p* < 0.005) and higher sensation-seeking scores (MSS: *t*(185) = −4.28, *p* < 0.001) than females, who, in turn, showed higher scores on anxiety (ANX: Welch’s *t*(107) = 3.03, *p* < 0.003). No significant gender differences were found in the level of education, overall cognitive functioning, and the remaining driving and personality measures.

### 3.2. Factor Analysis and Reliability of the MMPI-2-Based Sensation-Seeking Scale (MSS)

The items of the MSS were extracted from the MMPI-2, as reported by Viken et al. [119] by using the former version of the MMPI. Exploratory factor analysis (EFA) was performed on 16 items to investigate the factorial structure of the instrument. The Kaiser–Meyer–Olkin measure verifies a discrete sampling adequacy (KMO = 0.71), and Bartlett’s test of sphericity indicated that the correlation structure was adequate for the analysis (*χ*^2^ (120) = 372, *p* < 0.001). Six items showed factor loadings below the set cutoff (cutoff = 0.25) and were removed. The single-factor solution made by 10 items accounted for 17.5% of the variance, with factor loadings from 0.295 to 0.685. The best-fitting model provided an adequate fit to the data (*χ*^2^ (35) = 50.9, *p* < 0.04; TLI = 0.862, BIC = −132, RMSEA = 0.052, 90% CI = 0.012–0.078). Internal consistency for the unique factor of the MSS total score was also evaluated using Cronbach’s alpha. The MSS showed an internal consistency value of 0.66.

### 3.3. Multiple Regression Analyses

A series of multiple regression analyses was run considering the personality traits included in PSY-5 and IVPE-MMPI-based models as predictors of the driving measures. For each driving outcome (e.g., DT, MS, RS, ATAVT), four multiple regression models were created: two uncontrolled and two controlled for demographics. 

#### 3.3.1. Resilience of Attention (DT)

The first group of regression models were tested on the DT. The first comparison was performed between models uncontrolled for demographics. Effects are reported in Table 1a. R^2^ for the IVPE-MMPI model was equal to 0.11 (adj. *R*^2^ = 0.089) and for the PSY-5 model was equal to 0.03 (adj. *R*^2^ = 0.013). A lower AIC was found for the former (AIC = 1722.726) compared to the latter (AIC = 1738.693). Moreover, the models showed almost equal small effect sizes (Cohen’s *f*2 < 0.15). The PSY-5 model showed no significant effects of personality predictors. With respect to the IVPE-MMPI model, significant results were found for the effects of sensation seeking (*β* = 0.660, *p* < 0.001) and psychopathic deviation (*β* = 0.475, *p* < 0.012).

The second comparison was performed controlling for demographics in both the IVPE-MMPI model and the PSY-5 model. Significant and non-significant effects are reported in Table 1b. R^2^ for the IVPE-MMPI model was equal to 0.41 (adj. *R*^2^ = 0.39) and for the PSY-5 model was equal to 0.42 (adj. *R*^2^ = 0.39). A lower AIC was found for the latter (AIC = 1650.703) compared to the former (AIC = 1650.544). Moreover, the models showed almost equal large effect sizes (Cohen’s *f*2 > 0.60). Significant effects were found for age and level of education in both the IVPE-MMPI model (AGE: *β* = −25.869, *p* < 0.001; EDU: *β* = 1.741, *p* < 0.014) and the PSY-5 model (AGE: β = −27.550, *p* < 0.001; EDU: *β* = 1.589, *p* < 0.028).

#### 3.3.2. Reaction Speed (RS)

The first comparison was performed between models uncontrolled for demographics. Effects are reported in Table 2a. R^2^ for the IVPE-MMPI model was equal to 0.05 (adj. *R*^2^ = 0.03) and for the PSY-5 model was equal to 0.06 (adj. *R*^2^ = 0.03). A lower AIC was found for the former (AIC = 1770.433) compared to the latter (AIC = 1771.143). Moreover, the models showed almost equal small effect sizes (Cohen’s *f*2 < 0.15). The PSY-5 model showed no significant effects of personality predictors. With respect to the IVPE-MMPI model, a significant result was found for the effect of sensation seeking (*β* = 0.578, *p* < 0.007).

The second comparison was performed controlling for demographics (e.g., age, gender, and level of education) in both models. Effects are reported in Table 2b. R^2^ for the IVPE-MMPI model was equal to 0.16 (adj. *R*^2^ = 0.13) and for the PSY-5 model was equal to 0.20 (adj. *R*^2^ = 0.17). A lower AIC was found for the latter (AIC = 1745.271) compared to the former (AIC = 1753.562). Moreover, the models showed medium effect sizes (Cohen’s *f*2 = 0.15). Significant effects were found for age and gender in both the IVPE-MMPI model (AGE: *β* = −11.960, *p* < 0.009; GEN: *β* = 12.980, *p* < 0.003) and the PSY-5 model (AGE*: β* = −15.517, *p* < 0.001; GEN: *β* = 12.222, *p* < 0.002).

#### 3.3.3. Motor Speed (MS)

The first comparison was performed between models uncontrolled for demographics. Effects are reported in Table 3a. R^2^ for the IVPE-MMPI model was equal to 0.13 (adj. *R*^2^ = 0.11) and for the PSY-5 model was equal to 0.04 (adj. *R*^2^ = 0.01). A lower AIC was found for the former (AIC = 1730.416) compared to the latter (AIC = 1751.499). Moreover, the models showed almost equal small effect sizes (Cohen’s *f*2 < 0.15). The PSY-5 model showed no significant effects of personality predictors. With respect to the IVPE-MMPI model, significant results were found for the effects of sensation seeking (*β* = 0.756, *p* < 0.001) and anxiety (*β* = −0.651, *p* < 0.004).

The second comparison was performed controlling for demographics (e.g., age, gender, and level of education) in both models. Effects are reported in Table 3b. R^2^ for the IVPE-MMPI model was equal to 0.36 (adj. *R*^2^ = 34) and for the PSY-5 model was equal to 0.37 (adj. *R*^2^ = 0.34). A lower AIC was found for the former (AIC = 1677.486) compared to the latter (AIC = 1677.801). Moreover, the models showed almost large effect sizes (Cohen’s *f*2 > 0.35). Significant effects were found for age and gender in both the IVPE-MMPI model (AGE: *β* = −15.537*, p* < 0.001; GEN: *β* = 23.393, *p* < 0.001) and the PSY-5 model (AGE: *β* = −17.381, *p* < 0.001; GEN: *β* = 23.598, *p* < 0.001). Considering the PSY-5 model, a significant effect of introversion (*β* = −3.500, *p* < 0.048) emerged.

#### 3.3.4. Perceptual Speed (ATAVT)

The last group of regression models was tested on the ATAVT. The first comparison was performed between models uncontrolled for demographics. Effects are reported in Table 4a. R^2^ for the IVPE-MMPI model was equal to 0.08 (adj. *R*^2^ = 0.06) and for the PSY-5 model was equal to 0.01 (adj. *R*^2^ = 0.01). A lower AIC was found for the former (AIC = 1778.803) compared to the latter (AIC = 1793.477). Moreover, the models showed almost equal small effect sizes (Cohen’s *f*2 < 0.15). The PSY-5 model showed no significant effects of personality predictors. Considering the IVPE-MMPI model, a significant result was found for the effect of sensation seeking (*β* = 0.820, *p* < 0.001).

The second comparison was performed controlling for demographics (e.g., age, gender, and level of education) in both the IVPE-MMPI model and the PSY-5 model. Significant and non-significant effects are reported in Table 4b. R^2^ for the IVPE-MMPI model was equal to 0.21 (adj. *R*^2^ = 0.18) and for the PSY-5 model was equal to 0.20 (adj. *R*^2^ = 0.17). A lower AIC was found for the former (AIC = 1755.272) compared to the latter (AIC = 1760.332). Moreover, the models showed almost equal medium effect sizes (Cohen’s f2 > 0.15). Significant effects were found for age and level of education in both the IVPE-MMPI model (AGE: *β* = −18.100, *p* < 0.001; EDU: *β* = 1.857, *p* < 0.046) and the PSY-5 model (AGE: *β* = −18.592, *p* < 0.001; EDU: *β* = 2.144, *p* < 0.027).

## 4. Discussion

The present study aimed to compare the relationships between two MMPI-2-based models (IVPE-MMPI vs. PSY-5) and cognitive measures of the fitness to drive in a sample of young and adult drivers. Demographic characteristics (i.e., age, gender, and level of education) were also considered among the predictors. The study showed that demographic variables have a strong effect on cognitive prerequisites of driving, shadowing the contribution of personality factors (i.e., IVPE-MMPI and PSY-5 factors). Results showed that (i) age negatively affects all driving measures; (ii) gender significantly predicts a higher reaction and motor speed, in favor of males; and (iii) the level of education positively affects resilience of attention and perceptual speed. The group of adult participants showed poorer performance in all the driving subtasks than the younger participants. Moreover, male participants exhibited significantly lower visual and motor reaction times than females. Finally, the level of education predicted the accuracy of the participants’ performance in DT and ATAVT tasks, indicating higher levels of the fitness to drive. These results highlighted the crucial role of the demographic characteristics as determinants of the fitness to drive over the measures of personality. Regardless of demographic variables, the IVPE-MMPI-based model showed a better fit with all the driving measures than the PSY-5 model. As shown by the comparisons between the models’ AIC values, IVPE-MMPI-based personality traits showed a better predictive model on each driving prerequisites than PSY-5 traits. Results showed that (i) sensation seeking positively affects all the cognitive prerequisites; (ii) psychopathic deviation, as a measure of self-control, positively affects the resilience of attention; and (iii) anxiety, as a measure of emotional stability, negatively affects motor reaction speed. 

### 4.1. Effects of Demographic Variables

Previous studies have encouraged the consideration of age, gender, and level of education as key human characteristics in the prediction of risky driving [51] and collision rates [125]. The results of the present study showed negative effects of age on resilience of attention (Table 1b), reaction times (Table 2b and Table 3b), and perceptual speed (Table 4b). These results support previous evidence of the age-associated decline in cognitive, motor, and perceptual functions being important for driving, such as eyesight (e.g., diminished visual acuity and narrowed peripheral vision), reaction speed, perceptual speed, selective attention, and muscle strength [5,81,126,127,128,129]. Moreover, these results partially replicate evidence found by Nechtelberger et al. [77] in a sample of intoxicated drivers. By using the Vienna-DRIVESC package, the authors recently demonstrated that older age is linked to poor performance in eye–hand coordination, resilience of attention, reaction speed, perceptual speed, and short-term memory, reflecting decreased driving fitness. These age-related changes were attributed to a deterioration of biological systems [130] that lead to structural and functional changes in diverse brain regions (e.g., prefrontal cortex, cerebellar gray and white matter) [131,132,133]. Anstey et al. [7] summed up a series of age-related changes (e.g., increased physical frailty, decreased visual acuity, executive functioning decline, etc.) associated with crash risk and driving cessation in older drivers. 

The results of the present study revealed gender differences in simple reaction times favoring males. Male participants showed significantly higher reaction (Table 2b) and motor (Table 3b) speeds compared with female participants, connecting lower reaction times to higher levels of the fitness to drive. This result confirms that of Dykiert et al. [134], who found lower reaction times in males than in females in simple but not in complex reaction tasks. In the study of Karia et al. [135], males showed lower reaction times than females in both simple and complex visual reaction tasks. In terms of motor speed, several studies have demonstrated that the male gender is significantly associated with a lower response time [136,137,138]. These differences directly affected the simulated driving performance, resulting in slower brake reactions in female drivers [138]. In a large number of the above-cited studies, differences between males and females in reaction times were attributed to the differential hormonal action (e.g., testosterone exposure, antidiuretic hormone, insulin, etc.) that affects axonal and nerve conduction [139,140,141].

Moreover, a significant effect of the level of education was found on the measures of resilience of attention (Table 1b) and perceptual speed (Table 4b): with an increase in the number of years of education, the fitness to drive increased. Previous studies have demonstrated that education affects cognitive performance in different ways, directly by stimulating cognitive functions and brain development and indirectly through lifestyle choices, social interaction, types of occupation, and health behaviors [142,143]. Education offers enriching experiences that improve domain-general cognitive skills such as processing speed, working memory, reasoning, and cognitive control [144,145,146]. It has been argued that the effects of education on cognitive functioning are evident across the lifespan, influencing individual differences in cognitive skills that appear in adulthood and persist with aging [147]. A few studies have investigated the effect of the level of education on cognitive abilities that are crucial for the fitness to drive. From a behavioral perspective, a high level of education is found to be positively associated with an increased tendency to speed and to wear safety belts [148]. In a previous study aimed to investigate the role of cognitive abilities in predicting measures of the fitness to drive [81], we found similar results on resilience of attention and perceptual speed for the effect of the overall cognitive functioning but not for education. It is likely that education accounted for the same explained variance that was predicted by cognitive functioning on the same driving prerequisites. The significant correlation observed between the MoCA total score and the participants’ level of education supported this conclusion (Table S1). Overall, these results confirmed that education is a modifiable factor associated with cognitive functioning [149] that constitutes a strong cognitive component of safe driving [148]. 

### 4.2. Effects of Personality Traits Models

In the present study, both the personality models in use (i.e., IVPE-MMPI model and PSY-5 model) showed a small effect size in the prediction of driving cognitive prerequisites(Table 1a, Table 2a, Table 3a and Table 4a). As stated above, the effects of some personality traits lost their statistical significance when demographic variables were added among predictors. The results confirmed the idea that personality is a *distal* or a *secondary* human factor affecting the fitness to drive beyond more proximal factors (i.e., driving style and performance indices) [10]. It cannot be ignored that proximal factors are most affected by socio-demographic rather than personality factors [51]. The MMPI-2 personality traits explained a small amount of variance of the cognitive prerequisites moreover overshadowed by the effects of the demographic variables. In other words, these effects may have resulted in illusory relationships between personality and cognitive prerequisites largely explained by demographic factors, indeed. Given the distal role of personality in the models of driving behavior, these results may be attributable to (i) the smaller sensitivity of the measures of personality in discriminating the fitness to drive compared with other classes of variables (e.g., demographic, cognitive, behavioral) [10] and (ii) their susceptibility to response bias, such as simulating a socially desirable response to self-report data [150,151]. This bias affects particularly self-reported measures collected in the context of assessment procedures aimed at obtaining a personal advantage (e.g. driving or firearms license, child adoption competence) [152,153]. Nevertheless, significant results found in models neglecting for the contribution of demographic variables have also been discussed, as follows. 

First, the IVPE-MMPI model demonstrated a better fit to the data than the PSY-5 moedl. PSY-5 scales detect personality differences that affect clinical problems [105]. The better fit found for the IVPE-MMPI model could be explained with reference to the non-clinical condition of participants involved in this study. It is likely that PSY-5 scales, thought to detect personality traits that could be early markers of clinical problems in everyday life, were less specific in predicting driving outcomes. According to Nef et al. [67], the employment of standard personality questionnaires for the assessment of driving-related personality traits generally leads to non-significant results. Moreover, since the items of the personality scales included in the PSY-5 model in many cases can be referred to symptoms that are clearly recognizable even by unexperienced people [150,151,154], it is plausible to assume that these items were subjected to higher levels of self-deception or impression management than those included in the IVPE-MMPI model [150,151,154]. 

Significant results were found for the effects of sensation seeking on resilience of attention, reaction time, and perceptual speed. The higher the sensation seeking, the higher the performance, which reflects an increased fitness to drive. These results are quite new if compared with those reported in the literature that showed the detrimental role of sensation seeking on driving safety [14,59,61,62,63,64]. Sensation seeking has been frequently associated with unsafe driving outcomes such as driving violations and motor vehicle accidents [59,60]. Nonetheless, in a previous study by Nechtelberger et al. [77], sensation seeking was the only nonpredictive personality trait of the same prerequisites of the fitness to drive assessed in this study. Moreover, in the study by Sommer et al. [5], sensation seeking was not significantly associated with the measures of on-road driving performance. According to Sommer et al. [5], this could mean that sensation seeking negatively affects decisions made at the strategic level, whereas the criterion measure of driving fitness used in the present study entirely accounted both tactical and operational driving behaviors [5,6]. Moreover, it has been demonstrated that high sensation seekers exhibit enhanced responses to intense stimulation [48,155]. This could particularly affect the tactical and operational driving behaviors, especially when speed is higher and decision times decrease. A recent study showed that high sensation seekers perform better and quicker in a stressful physical task than low sensation seekers, releasing a significantly lower level of cortisol and showing less strain [155]. Furthermore, measures of sensation seeking have been found strongly linked to extreme sports participation [156]. The present results, showing positive effects of sensation seeking on cognitive and motor prerequisites of the fitness to drive, could also be explained by reference to the arousability of the noradrenergic system [56]. When exposed to intense stimulation, higher sensation seekers show increased central nervous activity, which allows them to react in a more efficient way than lower sensation seekers [48]. As a result, higher sensation seekers may show an accurate control of the vehicle, at least at an operational level, particularly in highly stimulating driving situations (e.g., driving on a single-lane costal road).

Psychopathic deviation, as a measure of self-control, showed significant effects on resilience of attention. The higher the PD score, the higher the traffic stress tolerance. This result seems to suggest that high self-control could negatively affect performance in complex reaction tasks. Previous studies have demonstrated that measures of self-control, extrapolated from the IVPE, fail in significantly predicting resilience of attention when compared with emotional instability [5,6,7,8,9,10,11,12,13,14,15,16,17,18,19,20,21,22,23,24,25,26,27,28,29,30,31,32,33,34,35,36,37,38,39,40,41,42,43,44,45,46,47,48,49,50,51,52,53,54,55,56,57,58,59,60,61,62,63,64,65,66,67,68,69,70,71,72,73,74,75,76,77]. Despite the PD scale being an MMPI-based measure more linked with self-control, it might be not suitable for the assessment of this construct. However, previous research demonstrated that some pathological traits are linked to over-focused selective attention (e.g., social dominance, fearlessness, etc.) [157]. Sadeh and Verona [158] found that high psychopathic personality traits were associated with reduced distractor processing in an attentional task compared to participants who scored lower in these traits. Moreover, psychopathic traits were associated with the tendency to take risks [158]. It is likely that the PD scale used in this study detected subthreshold psychopathology symptoms since it assesses markers of subclinical expression of the clinical disease. The fact that psychopathic personality traits have been associated with a reduced deep processing of irrelevant stimuli could partially explain the findings of this study observed in a non-clinical sample. 

Moreover, the results showed a significantly negative effect of anxiety, as a measure of emotional instability, on motor reaction times. A plethora of studies have reported that emotional instability is one of the driving-related personality traits that contribute to enhancing the prediction of the fitness to drive in young and adult drivers [5,19,60,61,62,77]. Sommer et al. [5] assessed the drivers’ personality by using the IVPE and employed a standardized driving test as a criterion of driving fitness. The authors found that emotional stability was the main variable among others personality traits (e.g., sensation seeking, social responsibility, and self-control) significantly associated with on-road driving performance. Vetter et al. [78] showed that social responsibility was the only personality trait that significantly predicted the driving performance in a sample of professional drivers. It seems that in professional drivers, emotional stability is not the main predictor of the measures of driving performance. The authors argued that their contrasting results to those found by Sommer et al. [5] were attributable to the methodology used: professional drivers were subjected to comprehensive personality assessment before being hired in their company, and candidates with less suitable personality profiles were excluded. Thus, social responsibility played a more important role in predicting the fitness to drive of professional drivers than emotional instability [78]. Moreover, the results presented in this study for the effect of anxiety are in line with those of Nechtelberger et al. [77], who found that high scores in emotional instability result from an increased physical/motor reaction time. Considering this evidence, we could argue that the anxiety scale coming from the MMPI-2 might be suitable for the assessment of the driver’s emotional instability. 

Finally, a significant effect of introversion (PSY-5) was found on motor reaction times, in addition to the effects of demographic variables. The higher the introversion score, the lower the motor reaction speed. Previous studies have reported extroversion-related differences in motor processing speed [159,160,161]. Extroverts showed lower reaction times in motor but not in visual reaction tasks (speed reaction) than introverts [159]. Introverts preferred accuracy over speed, whereas extroverts tended to respond faster regardless of accuracy [161]. Such extroversion-related differences in motor reaction speed were explained with a psychophysiological-behavioral experimental paradigm by Stahl and Rammsayer [161]. The authors demonstrated that introverts were faster in premotor processing than extroverts but slower in central and peripheral motor processing. This result suggests the potential usefulness of the introversion scale of the MMPI-2 in the prediction of motor speed connected to driving.

Regardless of the effects of the demographic group of variables, results suggested that measures of sensation seeking, anxiety (as a measure of emotional instability), psychopathic deviation (as a measure of self-control), and introversion, taken from the MMPI-2, could be valid predictors of driving fitness at the tactical and operational levels of driving behaviors [6]. 

Overall, this study suggested the paramount importance of not omitting the inclusion of demographic variables in the study of trait factors (i.e., personality, attitudes, cognition) associated with driving behavior. Socio-demographic factors should be controlled in the assessment of personality determinants for the fitness to drive to achieve more accurate predictive models, also isolating the real contribution of personality variables in predicting driving behavior. 

## 5. Conclusions

Personality traits have been considered a *must-have* domain of psychological assessment for the fitness to safe driving since the early 20th century [162]. To the best of our knowledge, this was the first research that employed two MMPI-2-based models to assess driving-related personality traits in predicting the fitness to drive. The present study could be helpful for professionals in driving mobility centers in the context of the assessment of the psychological fitness to drive, particularly for the driving license renewal of young and adult drivers. Moreover, the assessment of psychopathological personality traits associated with risky driving, through the PSY-5 scale, could be useful in the screening of professional drivers to be hired in transportation companies. Nonetheless, the present study provides a photograph with light and shadow on the role of personality as a significant predictor of the fitness to drive, suggesting its weak predictive value for cognitive prerequisites of safe driving when controlled for demographic variables.

Future research could include theoretical and methodological improvements. First, the predictive validity of other MMPI-2 scales (e.g., clinical, content, supplementary, etc.) in the evaluation of the psychological fitness to drive might be explored. Second, the social desirability bias and in general the effect of dissimulation should be controlled in self-reported personality measures, especially in the context of driving license revision/renewal. Third, the right-hand form administered for the subtest of perceptual speed (ATAVT) limits the generalization of the obtained results to countries with right-hand driving. Further research may find useful to compare both the left- and right-hand form with participants from countries with different traffic laws. Finally, other domains such as driving attitudes, values, and habits [163] might be introduced to improve the effectiveness of driving behavior and accident risk prediction, keeping the demographic variables constant. A fully multidimensional perspective on human factors related to driving behaviors should help scholars and policy makers to ensure a greater degree of safety for people *behind the wheel* and for all other fragile road users. 

## Figures and Tables

**Table 1:** Standardized beta coefficients. Standard errors and significance levels for predictors of the Determination Test (DT) variable. Adjusted R^2^ and Akaike Information Criterion (AIC) are also reported. Comparisons between models are presented uncontrolling for demographics (**a**) and considering their contribution (**b**).

(a) MODELS’ COMPARISON: DETERMINATION TEST									
	IVPE-MMPI					PSY-5			
	*β*	Std. Err.	*t*	*p*-Value		*β*	Std. Err.	*t*	*p*-Value
MSS	0.660	0.187	3.526	<0.001	AGGR	−0.162	0.219	−0.742	0.458
SR	0.129	0.210	0.614	0.539	PSYC	−0.491	0.277	−1.775	0.077
PD	0.475	0.188	2.526	0.012	DISC	0.301	0.196	1.532	0.127
ANX	−0.188	0.219	−0.856	0.393	NEGE	−0.022	0.247	−0.089	0.929
					INTR	0.101	0.207	0.489	0.625
Adj. R^2^	0.089					0.013			
ΔAdj. R^2^	0.076								
AIC	1722.726					1738.693			
**(b) MODELS’ COMPARISON: DETERMINATION TEST**									
	**IVPE-MMPI**					**PSY-5**			
	***β***	**Std. Err.**	***t***	***p*-Value**		***β***	**Std. Err.**	***t***	***p*-Value**
AGE	−25.869	3.477	−7.437	<0.001	AGE	−27.550	3.326	−8.283	< 0.001
GEN	−1.593	3.313	−0.481	0.631	GEN	−0.900	3.052	−0.295	0.768
EDU	1.741	0.701	2.482	0.014	EDU	1.589	0.718	2.212	0.028
MSS	0.112	0.174	0.642	0.522	AGGR	0.078	0.177	0.439	0.661
SR	0.038	0.173	0.219	0.827	PSYC	−0.340	0.218	−1.599	0.120
PD	0.175	0.157	1.112	0.267	DISC	−0.012	0.158	−0.077	0.938
ANX	−0.130	0.184	−0.704	0.482	NEGE	−0.028	0.196	0.142	0.887
					INTR	−0.003	0.163	−0.020	0.984
Adj. R^2^	0.390					0.393			
ΔAdj. R^2^	0.003								
AIC	1650.703					1650.544			

MMPI, Minnesota Multiphasic Personality Inventory; IVPE, Inventory of Driving-related Personality Traits; PSY-5, Personality Psychopathology Five; MSS, MMPI-based sensation-seeking scale; SR, social responsibility; PD, psychopathic deviation; ANX, anxiety; AGGR, aggressiveness; PSYC, psychoticism; DISC, disconstraint; NEGE, negative emotionality; INTR, introversion; AGE, age; GEN, gender; EDU, education.

**Table 2:** Standardized beta coefficients. Standard errors and significance levels for predictors of the reaction speed (RS) variable. Adjusted R^2^ and Akaike Information Criterion (AIC) are also reported. Comparisons between models are presented uncontrolling for demographics (**a**) and considering their contribution (**b**).

(a) MODELS’ COMPARISON: REACTION SPEED									
	IVPE-MMPI					PSY-5			
	*β*	Std. Err.	*t*	*p*-Value		*β*	Std. Err.	*t*	*p*-Value
MSS	0.578	0.212	2.721	0.007	AGGR	0.138	0.239	0.580	0.536
SR	−0.074	0.238	−0.312	0.755	PSYC	−0.134	0.302	−0.443	0.659
PD	0.167	0.213	0.784	0.434	DISC	0.312	0.215	1.453	0.148
ANX	−0.119	0.249	−0.476	0.634	NEGE	−0.356	0.269	−1.320	0.189
					INTR	−0.347	0.226	−1.537	0.126
Adj. R^2^	0.033					0.034			
ΔAdj. R^2^	0.001								
AIC	1770.433					1771.143			
**(b) MODELS’ COMPARISON: REACTION SPEED**									
	**IVPE-MMPI**					**PSY-5**			
	***β***	**Std. Err.**	***t***	***p*-Value**		***β***	**Std. Err.**	***t***	***p*-Value**
AGE	−11.960	4.578	−2.612	0.009	AGE	−15.517	4.284	−3.621	<0.001
GEN	12.980	4.362	2.975	0.003	GEN	12.222	3.931	3.109	0.002
EDU	1.772	0.923	1.919	0.056	EDU	1.017	0.925	1.099	0.273
MSS	0.065	0.229	0.286	0.775	AGGR	0.222	0.228	0.974	0.331
SR	−0.098	0.227	−0.434	0.664	PSYC	0.025	0.281	0.092	0.917
PD	0.052	0.207	0.249	0.803	DISC	0.086	0.253	−1.568	0.118
ANX	0.079	0.243	0.327	0.743	NEGE	−0.395	0.252	−1.568	0.118
					INTR	−0.389	0.210	−1.849	0.066
Adj. *R*^2^	0.130					0.172			
ΔAdj. *R*^2^	0.042								
AIC	1753.562					1745.271			

**Table 3:** Standardized beta coefficients. Standard errors and significance levels for predictors of the motor speed (MS) variable. Adjusted R^2^ and Akaike Information Criterion (AIC) are also reported. Comparisons between models are presented uncontrolling for demographics (**a**) and considering their contribution (**b**).

(a) MODELS’ COMPARISON: MOTOR SPEED									
	IVPE-MMPI					PSY-5			
	*β*	Std. Err.	*t*	*p*-Value		*β*	Std. Err.	*t*	*p*-Value
MSS	0.756	0.191	3.958	<0.001	AGGR	−0.055	0.226	−0.243	0.808
SR	−0.015	0.214	−0.074	0.940	PSYC	−0.306	0.287	−1.068	0.286
PD	0.375	0.191	1.955	0.051	DISC	0.365	0.203	1.795	0.074
ANX	−0.651	0.224	−2.907	0.004	NEGE	0.307	0.256	1.201	0.231
					INTR	−0.315	0.214	−1.472	0.143
Adj. R^2^	0.113					0.012			
ΔAdj. R^2^	0.101								
AIC	1730.416					1751.499			
**(b) MODELS’ COMPARISON: MOTOR SPEED**									
	**IVPE-MMPI**					**PSY-5**			
	***β***	**Std. Err.**	***t***	***p*-Value**		***β***	**Std. Err.**	***t***	***p*-Value**
AGE	−15.537	3.735	−4.159	<0.001	AGE	−17.381	3.578	−4.858	<0.001
GEN	23.393	3.559	6.572	<0.001	GEN	23.598	3.282	7.189	<0.001
EDU	1.234	0.753	1.637	0.103	EDU	1.161	0.773	1.503	0.135
MSS	0.042	0.187	0.226	0.822	AGGR	0.001	0.191	0.003	0.998
SR	−0.003	0.185	−0.017	0.986	PSYC	−0.071	0.235	−0.302	0.763
PD	0.261	0.169	1.548	0.123	DISC	0.076	0.170	0.444	0.658
ANX	−0.319	0.198	−1.613	0.108	NEGE	0.207	0.211	0.984	0.327
					INTR	−0.350	0.176	−1.991	0.048
Adj. R^2^	0.342					0.344			
ΔAdj. R^2^	0.002								
AIC	1677.486					1677.801			

**Table 4:** Standardized beta coefficients. Standard errors and significance levels for predictors of the Adaptive Tachistoscopic Traffic Perception Test (ATAVT) variable. Adjusted R^2^ and Akaike Information Criterion (AIC) are also reported. Comparisons between models are presented uncontrolling for demographics (**a**) and considering their contribution (**b**).

(a) MODELS’ COMPARISON: PERCEPTUAL SPEED									
	IVPE-MMPI					PSY-5			
	*β*	Std. Err.	*t*	*p*-Value		*β*	Std. Err.	*t*	*p*-Value
MSS	0.820	0.217	3.772	<0.001	AGGR	−0.240	0.253	−0.946	0.345
SR	0.194	0.244	0.796	0.426	PSYC	−0.192	0.320	−0.598	0.550
PD	0.132	0.218	0.605	0.546	DISC	0.255	0.227	1.120	0.264
ANX	−0.235	0.255	−0.921	0.358	NEGE	−0.078	0.286	−0.274	0.784
					INTR	0.126	0.239	0.528	0.598
Adj *R*^2^	0.060					0.011			
ΔAdj *R*^2^	0.049								
AIC	1778.803					1793.477			
**(b) MODELS’ COMPARISON: PERCEPTUAL SPEED**									
	**IVPE-MMPI**					**PSY-5**			
	***β***	**Std. Err.**	***t***	***p*-Value**		***β***	**Std. Err.**	***t***	***p*-Value**
AGE	−18.100	4.599	−3.936	<0.001	AGE	−18.592	4.460	−4.168	<0.001
GEN	1.720	4.328	0.393	0.695	GEN	5.401	4.092	1.320	0.189
EDU	1.857	0.927	2.002	0.046	EDU	2.144	0.963	2.226	0.027
MSS	0.360	0.230	1.567	0.118	AGGR	−0.122	0.237	−0.514	0.607
SR	0.124	0.228	0.543	0.588	PSYC	−0.038	0.293	−0.131	0.896
PD	−0.080	0.208	−0.387	0.699	DISC	0.013	0.212	0.062	0.950
ANX	−0.157	0.244	−0.642	0.521	NEGE	−0.043	0.262	−0.164	0.870
					INTR	0.071	0.219	0.328	0.743
Adj. *R*^2^	0.184					0.169			
ΔAdj. *R*^2^	0.015								
AIC	1755.272					1760.332			

## Data Availability

The data presented in this study are available in the Open Science Framework-OSF at 10.17605/OSF.IO/84H5X, reference number https://osf.io/84h5x.

## Supplementary Materials

The following are available online at https://www.mdpi.com/2076-3425/11/3/313/s1: Table S1: Correlation matrix between the variables and reliability coefficients.
