# Supplementary material for: The Impact of Two MMPI-2-Based Models of Personality in Predicting Driving Behavior. Can Demographic Variables Be Disregarded?
Source: Brain Sci. 2021 Mar 2;11(3):313. doi: 10.3390/brainsci11030313 (PMC8000114; doi:10.3390/brainsci11030313)
Supplement: Supplementary file 1 [file brainsci-11-00313-s001.pdf]

Table S1. Correlation matrix between the variables. Mean (M) and Standard Deviation (SD) for each variable; reliability coefficients are reported. *p< 0.05, **p< 0.01, ***p< 0.001.
AGE=age in years; GEN=gender of participants; EDU=years of education; MOCA=MoCA corrected score; AGGR=Aggression; PSYC=Psychoticism; DISC=Discontraint; NEGE=Negative Emotionality; INTR=Introversion; MSS=MMPI-based Sensation Seeking Scale; RE=Social Responsibility; PD=Psychopathic Deviation; ANX=Anxiety; DT=Schuhfried Vienna "Determination Test" score; MS=Schuhfried Vienna "Motor Speed"; RS=Schuhfried Vienna "Reaction Speed"; ATAVT=Schuhfried Vienna "Perceptual speed" score.

	AGE	*GEN*	*EDU*	*MOCA*	*AGGR*	*PSYC*	*DISC*	*NEGE*	*INTR*	*TMSS*	*RE*	*PD*	*ANX*	*DT*	*MS*	*RS*	*ATAVT*	*M*	*SD*	*Chronbach's α*
AGE	-																	37.6	16.6	-
GEN	−0,03	-																-	-	-
EDU	−0,44 ***	−0,07	-															12.3	2.30	-
MOCA	−0,25 ***	−0,01	0,32 ***	-														24.9	2.94	0.702
AGGR	0,12	0,12	0,04	0,01	-													53.0	10.3	0.618
PSYC	0,06	0,03	−0,12	−0,07	0,41 ***	-												55.6	9.31	0.722
DISC	−0,10	0,12	0,02	0,02	0,53 ***	0,39 ***	-											51.4	11.2	0.746
NEGE	0,03	0,09	−0,16 *	−0,13	0,30 ***	0,66 ***	0,27 ***	-										56.7	10.2	0.850
INTR	−0,08	-0,03	−0,09	−0,03	−0,15 *	0,11	−0,08	0,25 ***	-									50.7	9.36	0.740
TMSS	−0,40 ***	0,30 ***	0,20 **	0,14	0,09	−0,04	0,17	−0,05	0,04	-								57.7	9.79	0.656
SR	0,15 *	−0,02	0,01	0,00	−0,09	−0,11	−0,12 *	−0,08	−0,04	−0,29 ***	-							48.8	9.83	0.761
PD	−0,26 ***	−0,12	0,07	0,14	0,10	-0,02	0,07	0,08	0,10	0,18 *	−0,43 ***	-						54.1	11.4	0.826
ANX	−0,09	−0,23 ***	0,00	−0,06	−0,03	0,04	0,01	0,09	0,07	0,09	−0,43 **	0,53 ***	-					56.0	9.75	0.854
DT	−0,62 ***	−0,01	0,41 ***	0,43 ***	−0,08	−0,16 *	0,02	−0,10	0,02	0,28 ***	−0,09	0,20 **	0,04	-				65.2	24.9	0.962
MS	−0,39 ***	0,45 ***	0,22 **	0,20 **	0,07	0,01	0,15 *	0,06	−0,10	0,30 ***	−0,06	0,09	−0,13	0,35 ***	-			48.5	25.8	0.979
RS	−0,31 ***	0,22 **	0,23 **	0,19 **	0,08	−0,07	0,11	−0,14	−0,17 *	0,22 **	−0,10	0,10	0,03	0,40 ***	0,53 ***	-		52.5	27.5	0.919
ATAVT	−0,41 ***	0,08	0,31 ***	0,39 ***	−0,07	−0,07	0,02	−0,06	0,03	0,26 ***	0,00	0,03	−0,06	0,49 ***	0,31 ***	0,34 ***	-	50.7	28.5	0.623
